# Supplementary material for: Jahn-Teller-induced femtosecond electronic depolarization dynamics of the nitrogen-vacancy defect in diamond
Source: Nat Commun. 2016 Nov 16;7:13510. doi: 10.1038/ncomms13510 (PMC5116094; doi:10.1038/ncomms13510)
Supplement: Supplementary Information — Supplementary Figures 1-10, Supplementary Methods and Supplementary References [file ncomms13510-s1.pdf]

Supplementary Figures

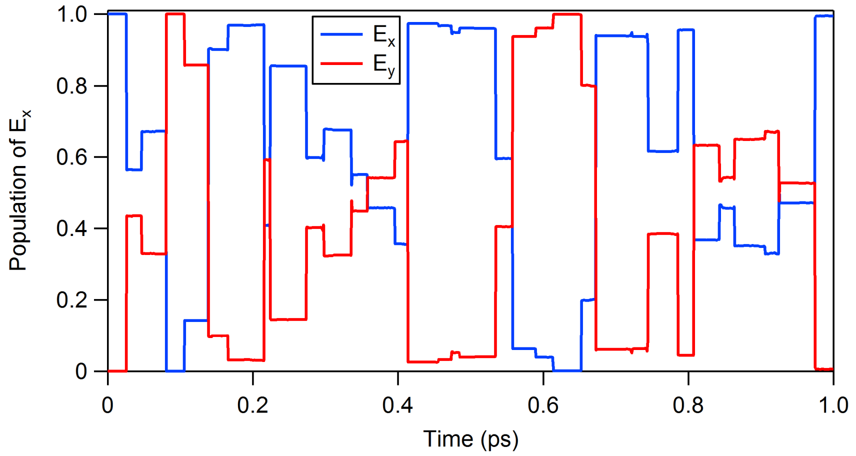

Supplementary Figure 1. Single trajectory of the orbital mixing dynamics.

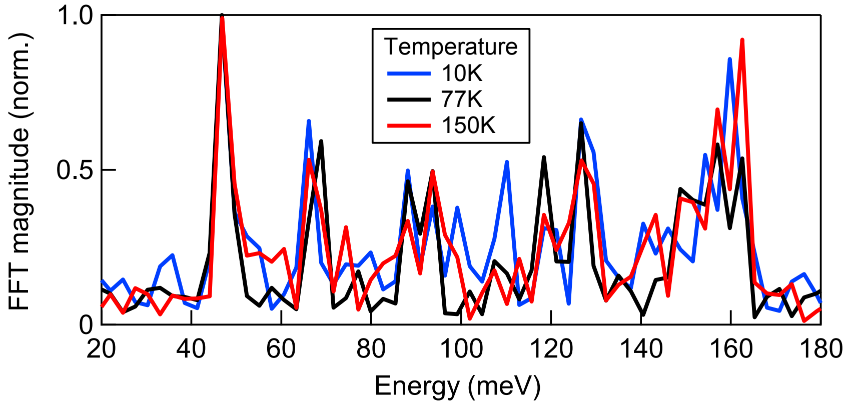

Supplementary Figure 2. Averaged normalized FFT magnitude of the orbital trajectories for 10K, 77K and 150 K.

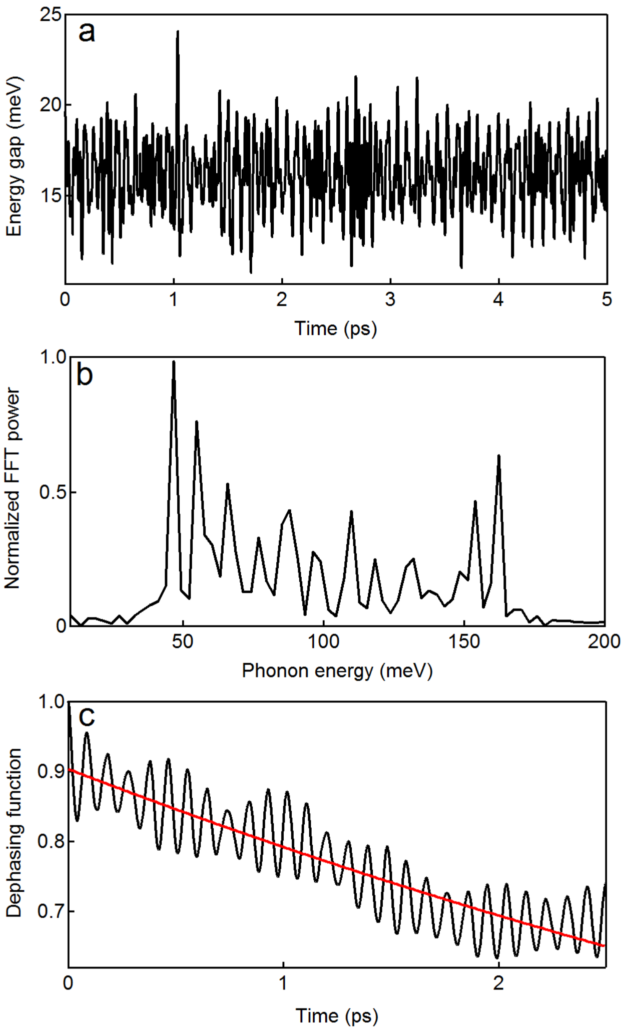

Supplementary Figure 3. Excited-state AIMD simulation results at 10 K. (a) Phonon-induced fluctuation of the energy gap between the two adiabatic surfaces of the ^3E excited state. **(b)** The FFT power spectrum showing the frequencies of phonon modes that induce the energy gap fluctuations. **(c)** The calculated dephasing function, together with an exponential fit that yields a time constant τ_2 of 8 ps.

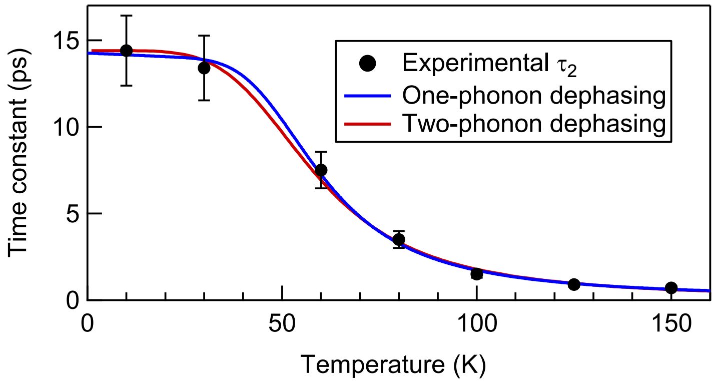

Supplementary Figure 4. Fits of the temperature dependence of the experimental τ_2 values of to the one-phonon and two-phonon dephasing model.

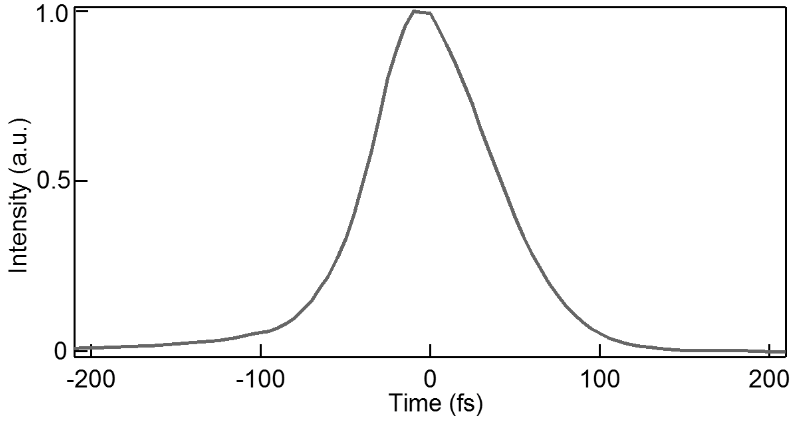

Supplementary Figure 5. Cross-correlation between the narrowband pump and broadband probe pulse yields an instrument response function with a FWHM of 80 fs.

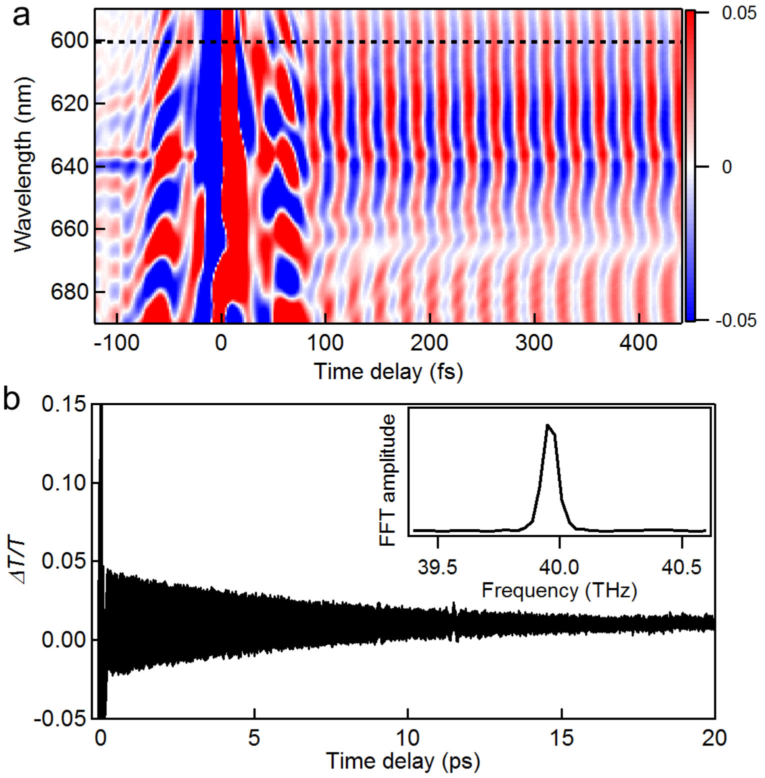

Supplementary Figure 6. Dynamics of the coherent LO phonon as excited by sub-10 fs pulses. (a) Spectrally resolved differential transmission signal showing the coherent LO phonon associated with the diamond lattice of the NV^- sample. **(b)** The temporal line-out at 600 nm (dashed line in **a**) showing the long dephasing of the coherent phonon. The inset shows the Fourier transform of the time trace, which yields the LO phonon frequency of 39.95 THz.

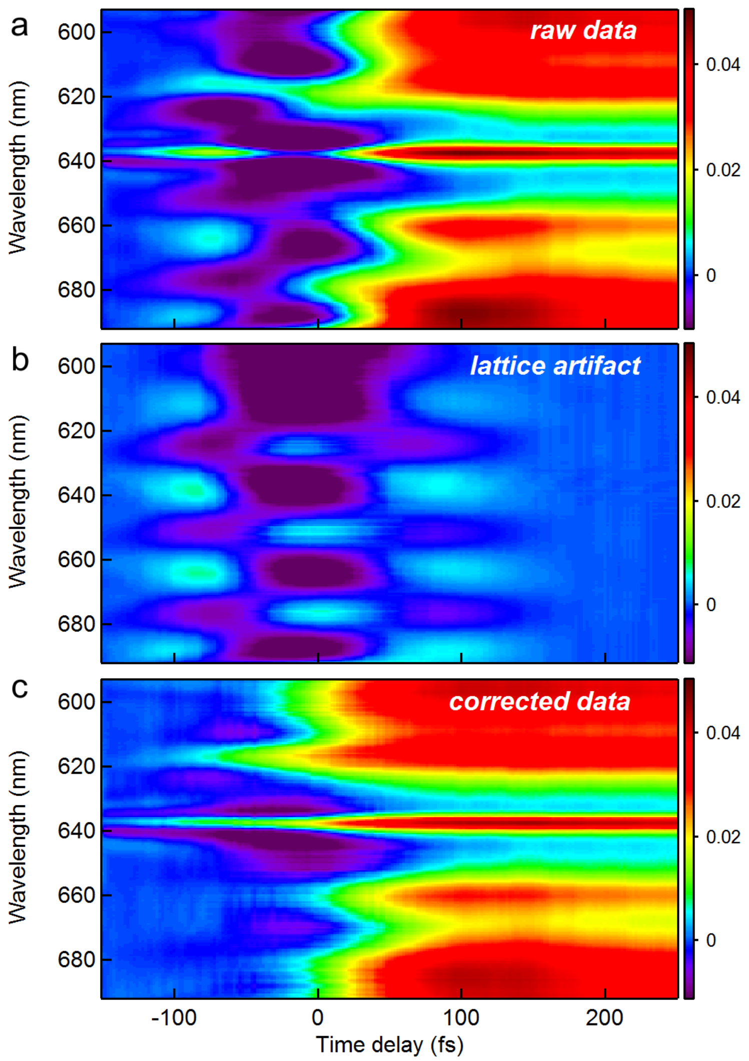

Supplementary Figure 7. Removal of the artifact generated by the diamond lattice. (a) The differential transmission response of the NV^- center with artifactual contributions from the diamond substrate. **(b)** The differential transmission signal of the pure diamond sample. **(c)** The data after removal of contributions from the substrate artifact, revealing the pure NV^- response.

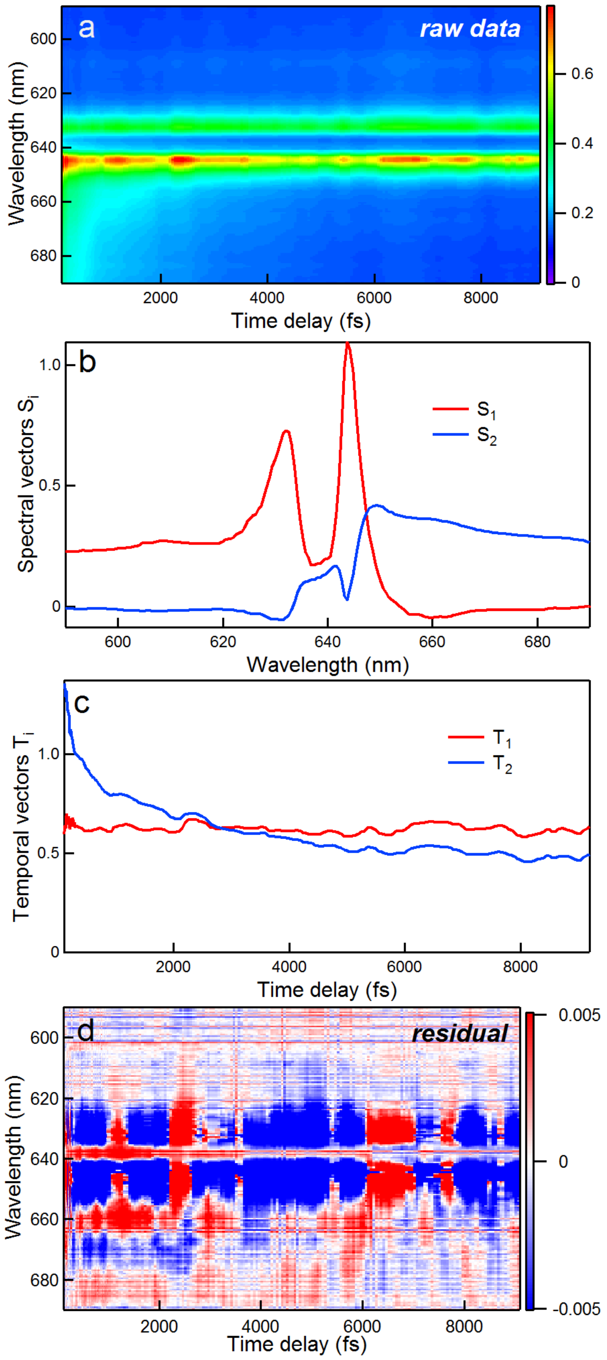

Supplementary Figure 8. Processing of anisotropic signal by multivariate curve resolution analysis. (a) Raw data **(b)** Spectral vectors **(c)** Temporal vectors and **(d)** Error matrix.

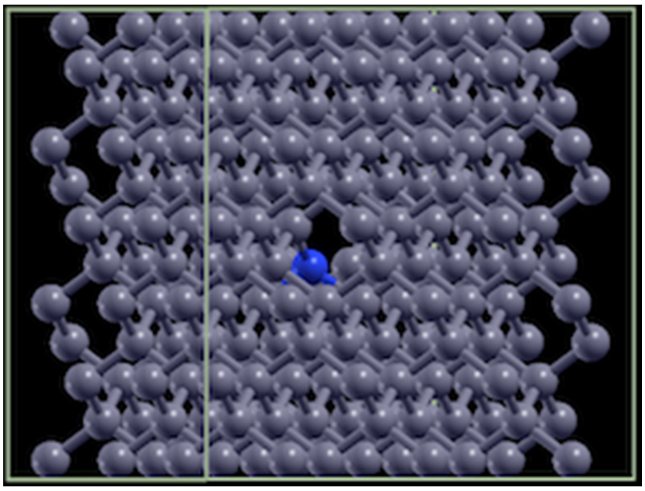

Supplementary Figure 9. Optimized structure of a NV-diamond at 0 K. One carbon atom next to the nitrogen atom (colored by blue) is removed from the diamond structure to make a vacancy.

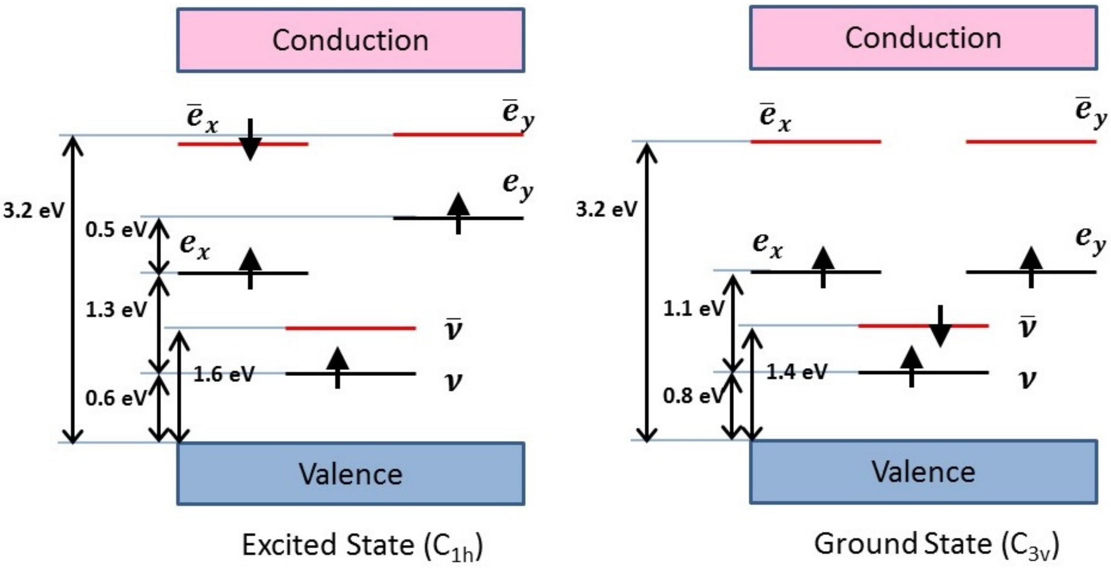

Supplementary Figure 10. Optimized electronic structures of the C_{1h} and C_{3v} symmetry corresponding to the excited and ground states, respectively. The excited $\boldsymbol{E}_{\mathrm{x}}$ and $\boldsymbol{E}_{\mathrm{y}}$ states are degenerated with the C_{3v} symmetry, while the degeneracy of the two excited states is resolved with the C_{1h} symmetry.

Supplementary methods

In the following, we provide detailed descriptions of the experimental and theoretical methods.

Experimental

We use two ultrafast transient absorption setups, both based on amplified Ti:Sapphire lasers: a broadband setup that furnishes probe pulses spanning 550 – 750 nm and wavelength-tunable narrowband pump pulses (10-nm bandwidth), and a two-color setup that uses tunable narrowband pulses (10-nm bandwidth) for both the pump and probe pulse. The narrow pump pulse bandwidth of both setups allows the selective excitation of the zero-phonon line (ZPL) at 637 nm. The sample was mounted in a liquid-nitrogen cooled cryostat (broadband setup, 77 – 300 K, Oxford Instruments) or a closed-cycle helium cooled cryostat (two-color setup, 10 – 300 K, Janis Research Company).

The broadband transient absorption setup is pumped by a Ti:Sapphire amplified laser (Coherent Legend Elite Duo-USX) that operates at a repetition rate of 1 kHz and delivers nominally 25-fs pulses with an average power of 5 W. Spectral broadening by self-phase modulation in a helium-filled hollow-core fiber followed by chirped mirror compression furnishes <5-fs pulses with wavelength spanning ~550 – 950 nm. Subsequent multiple reflections of the beam off of a pair of visible dielectric mirrors (CVI TLM-0-650-1037) results in a reduced spectral range of ~550 – 750 nm. The output pulse has a duration of <10 fs, as measured by auto-correlation in a BBO crystal. About 10% of the pulse energy is split off and used as the probe pulse. The transmitted probe pulse is detected by a 1024-pixel photodiode array (Entwicklungsbüro Stresing). The remaining light is utilized as the pump pulse, after further narrowing its spectrum by an optical bandpass filter with a passband of 10-nm

FWHM and center wavelength of 640 nm. Angle tuning of the bandpass filter yields pulses with a center wavelength of 637 nm.

Spectral filtering of the pump pulse serves several purposes. First, it suppresses the generation of coherent LO phonons of the diamond lattice via impulsive stimulated Raman excitation[1] (Supplementary Fig. 6), which would otherwise obscure the ultrafast dynamics intrinsic to the NV^- defect. Second, the 10-nm bandwidth allows the selective excitation of the zero-phonon line (ZPL) transition at 637 nm. Lastly, the spectral overlap between excitation and probe pulse is minimized, which in turn eliminates artifacts induced by sample scatter in the region of spectral overlap, which would otherwise distort the signal at early time delays. This artifact is readily apparent in Supplementary Fig. 6, collected with broadband, degenerate pump and probe pulses. The spectral filtering of the pump pulse comes at the expense of an increased pulse duration of the pump pulse. Nevertheless, the cross-correlation between pump and probe pulse is 80 fs (Supplementary Fig. 5), which remains sufficiently short to resolve the various ultrafast dynamics observed in this work.

The two-color pump-probe setup is based on an amplified Ti:Sapphire laser system (Spitfire Ace, Spectra Physics) that operates at a repetition rate of 1 kHz and delivers 70-fs pulses with an average power of 5 W. The output is used to pump two optical parametric amplifiers (TOPAS), where the first OPA delivers the excitation pulse at 637 nm and the second OPA the probe pulse at a wavelength of 660 nm. After transmission through the sample, the pump beam is removed using a suitable longpass filter and the probe beam is detected with a photodiode that is connected to a lock-in amplifier.

Data analysis

Elimination of artifacts arising from the diamond substrate

The pump-probe signal contains intricate dynamics around time zero that are not related to the NV^- defects but originate from nonlinear optical effects of the diamond substrate. Fortunately, akin to the ubiquitous solvent artifacts known in condensed phase time-resolved spectroscopy, these artifactual contributions to the signal can be effectively eliminated by subtracting the response of a Type IIa highly pure diamond sample (Element Six) from the NV^- response to yield the pure NV^- dynamics. Exemplary spectra for this procedure are shown in Supplementary Fig. 7. By subtracting the pure diamond response (Supplementary Fig. 7a) from the raw NV^- data (Supplementary Fig. 7b), with precise manual adjustments of time delay and magnitude of the substrate artifact, its influence on the NV^- dynamics can be removed almost completely (Supplementary Fig. 7c).

Calculating the polarization anisotropy

The ultrafast dynamics of the NV^- defect are reconstructed from two sets of differential transmission measurements – one in which the pump and probe beams have parallel polarizations ($S^{\parallel}$), and another one in which the polarizations are perpendicular ($S^{\perp}$). The differential transmission signal is defined as $S^{\mathrm{i}}(\lambda, t) = \left[T_{\mathrm{on}}^{\mathrm{i}}(\lambda, t) - T_{\mathrm{off}}^{\mathrm{i}}(\lambda, t)\right]/T_{\mathrm{off}}^{\mathrm{i}}(\lambda, t)$, where i denotes the polarization index (∥ or ⊥), λ is the probe wavelength, t is the time delay, and T_{on} (T_{off}) corresponds to the sample transmission in the presence (absence) of the photoexcitation pump pulse. Note that the measurements are performed on an ensemble of NV^- centers. The signals recorded with different relative polarizations between the pump and probe pulses can be further combined to yield the polarization anisotropy, given as $S_{\mathrm{aniso}} =$

$(S^{\parallel} - S^{\perp})/(S^{\parallel} + 2S^{\perp})$. S_{aniso} reflects the degree of orbital alignment and is therefore exquisitely sensitive to the dynamics of the ^3E orbital doublet, such as decoherence and population transfer.

Singular value decomposition

We use multivariate curve resolution (MCR) based on singular value decomposition (SVD) to identify the signal components in the anisotropic data and to further eliminate contributions from artifacts[2,3]. This technique decomposes the spectrally and time-resolved dataset into a linear combination of components with distinct temporal and spectral behavior. To this end, the spectrally and time-resolved plot is treated as a matrix D that can be decomposed into n components as

$$D = \sum_{\mathrm{i}}^{\mathrm{n}} T_{\mathrm{i}} S_{\mathrm{i}}^{T} + E, \tag{1}$$

where T_{i} and S_{i} are vectors representing the temporal and spectral characteristics of the i-th component, and E is the error matrix that contains the residual variation of the data. Signal decomposition methods such as the one applied here are commonly used to identify patterns within complex two-dimensional signals, such as the evolution of chemical species during a reaction as monitored by spectroscopic techniques[4].

The raw data of the anisotropic signals (Supplementary Fig. 8a) can each be decomposed into two components, yielding the spectral vectors S_1 and S_2 (Supplementary Fig. 8b) and the corresponding temporal vectors T_1 and T_2 (Supplementary Fig. 8c). The error matrices E, i.e., the residual, do not show any long-range temporal and spectral correlations (Supplementary Fig. 8d). In other words, the removed noise that is contained in E is largely uncorrelated and all significant dynamics have thus been captured by the dual-component S_{i} and T_{i} vectors. Note that MCR is applied only to data acquired at time delays larger than 100

fs in order to minimize the effect of the coherent response around time zero on the signal decomposition.

As can be seen from the contour plot of Supplementary Fig. 8a, the anisotropy exhibits a decay at the ZPL and on the red side of it, where stimulated emission occurs, whereas the anisotropy is constant on the blue side of the ZPL, where the signal is dominated by ground-state bleaching. The spectral and temporal vectors obtained from singular value decomposition is consistent with this result. The first component captures the constant anisotropy part on the ground-state-bleaching side, whereas the second component captures the biexponential decay on the stimulated emission side. The anomalously large anisotropy values adjacent to the ZPL transition are presumably artifacts that arise from the vanishing $\Delta T/T$ signal in those spectral regions, which cause errors in the anisotropy values to be greatly amplified.

Fitting of τ_2

In addition to the one-phonon dephasing model described in the main text, τ_2 can also be fitted to a two-phonon dephasing model. A temperature dependence similar to that observed for τ_2 has been reported for electronic dephasing rates inferred from single-NV^--center photoluminescence excitation linewidth measurements[5], albeit involving dephasing times that are three orders of magnitude longer than those observed here (10^1 ns vs. 10^1 ps at 10 K). The previously observed temperature-dependent depolarization was attributed to population transfer via two-phonon Raman scattering between the strain-split E_x and E_y states. According to this model, which assumes the Debye phonon density of states, the dephasing rate is given by[6]

$$\Gamma(T) = \Gamma_0 + CT^5 \int_0^{\hbar\omega_D/k_B T} \frac{x^4 e^x}{(e^x - 1)^2}\, dx\,, \qquad (2)$$

where Γ_0 is the temperature-independent offset, C is the coefficient that characterizes the phonon density of states and electron-phonon coupling strength, and $\hbar\omega_D$ is the Debye energy. Fitting the experimental dephasing rates $\Gamma(T) = 1/\tau_2(T)$ to Eq. (S2) yields $\Gamma_0 = 0.069 \pm 0.006$ ps^{-1} and $\hbar\omega_D = 35 \pm 4$ meV. This fit is shown in Supplementary Fig. 4, together with the fit to the one-phonon dephasing model. The satisfactory agreement suggests that the two-phonon Raman scattering process could be operative even for the picosecond dephasing times that are observed here. We note that photon echo spectroscopy performed on a spectral feature located on the high-energy side of the ZPL yielded a similar T^5-dependence for the dephasing times, although the origin of the high-energy spectral feature remains unknown[7].

Simulations

We performed real-time atomistic simulations for the anisotropy decay of an initially created dipole moment in an NV-diamond. A photoexcited state on one of two adiabatic states, E_x or E_y, which is close to the conical intersection, undergoes non-adiabatic (NA) transitions between E_x and E_y around the conical intersection. Mixing of the initially excited state created on the one state with the other state causes the anisotropy decay.

The electronic structures of both ground state and excited state as well as adiabatic molecular dynamics (MD) are obtained with the VASP software package, using the PBE density functional and projector-augmented-wave pseudopotentials. The geometry of the NV-diamond composed of 1 nitrogen atom and 214 carbon atoms (a 3×3×3 cubic supercell with length being 10.719 Å), shown in Supplementary Fig. 9, is fully optimized at zero temperature[6]. To achieve the negatively-charged NV^- center, a single electron is added to the electron occupancy. The ground state has C_{3v}

symmetry structure and $^3A_2$ orbital state. As for the excited-state optimization, we started from a lower symmetry atomic arrangement and forced the excitation of the down-spin electron from $\overline{v}$ to E_{x} orbital then fully relaxed the whole NV-diamond structure. The optimized excited-state structure has the symmetry of C_{1h} point group and its NV⁻ center is in the ^3E orbital state (Supplementary Fig. 10).

The NV ground-state of C_{3v} symmetry is then heated up to each temperature ranging from 10 K to 300 K by repeated velocity rescaling, and a 5 ps microcanonical trajectory at each temperature is calculated on the ground electronic state using the Verlet algorithm with a 1 fs time-step. This means that the atomic vibrational motions are treated using classical mechanics. A few thousands of initial conditions are sampled from the long MD trajectory to initiate the photoexcited NA dynamics.

The NA dynamics simulations for the NV-diamond are performed with adiabatic Kohn-Sham (KS) bases[8-10]. The diabatic KS orbitals, $\varphi_{\mathrm{p}}(\boldsymbol{r}, t)$, are time-evolved by the standard time-dependent KS equations

$$i\hbar \frac{\partial \varphi_{\mathrm{p}}(\boldsymbol{r}, t)}{\partial t} = H\big(\varphi(\boldsymbol{r}, t)\big)\varphi_{\mathrm{p}}(\boldsymbol{r}, t), \quad p = 1, \dots, N_{\mathrm{e}} \tag{3}$$

where N_{e} is the number of electrons. Each p-equation is coupled through the nonlinear dependence of the Hamiltonian H on the electron density. Expanding the diabatic KS orbitals $\varphi_{\mathrm{p}}(\boldsymbol{r}, t)$ by the adiabatic KS orbital basis $\tilde{\varphi}_{\mathrm{k}}(\boldsymbol{r}; \boldsymbol{R})$,

$$\varphi_{\mathrm{p}}(\boldsymbol{r}, t) = \sum_{k=1}^{N_{\mathrm{e}}} c_{\mathrm{pk}}(t) |\tilde{\varphi}_{\mathrm{k}}(\boldsymbol{r}; \boldsymbol{R})\rangle, \tag{4}$$

transforms eq. (S3) into an equation for the expansion coefficients

$$i\hbar \frac{\partial c_{\mathrm{pk}}(t)}{\partial t} = \sum_{k=1}^{N_{\mathrm{e}}} c_{\mathrm{pm}}(t)(\varepsilon_{\mathrm{m}}\delta_{\mathrm{km}} - i\hbar \boldsymbol{d}_{\mathrm{km}} \cdot \dot{\boldsymbol{R}}). \tag{5}$$

The adiabatic KS orbital $\tilde{\varphi}_{\mathrm{k}}(\boldsymbol{r};\boldsymbol{R})$ and the eigen energy ε_{k} are obtained by the time-domain density functional theory for atomic positions at each moment along the MD trajectory. The NA coupling to cause transitions between adiabatic states,

$$\boldsymbol{d}_{\mathrm{km}} \cdot \dot{\boldsymbol{R}} = \langle \tilde{\varphi}_{\mathrm{k}}(\boldsymbol{r};\boldsymbol{R}) | \nabla_{\mathbf{R}} | \tilde{\varphi}_{\mathrm{m}}(\boldsymbol{r};\boldsymbol{R}) \rangle \cdot \dot{\boldsymbol{R}} = \left\langle \tilde{\varphi}_{\mathrm{k}}(\boldsymbol{r};\boldsymbol{R}) \middle| \frac{\partial}{\partial t} \middle| \tilde{\varphi}_{\mathrm{m}}(\boldsymbol{r};\boldsymbol{R}) \right\rangle, \tag{6}$$

stems from the dependence of the adiabatic KS orbitals on the phonon dynamics $\boldsymbol{R}(t)$, and represents electron-phonon interactions. Since the NA coupling is proportional to the nuclear velocity $\dot{\boldsymbol{R}}$, NA transitions would not occur under the Born-Oppenheimer approximation with stationary atoms.

In our real-time simulation for the anisotropy decay of an initially created dipole moment, we only calculate the adiabatic KS orbitals, their eigen energies and the NA coupling related to down-spin states because both the E_{x} and E_{y} states only possess down spin. The initial expansion coefficient of the E_{x} orbital is set as unity. The NA mixing dynamics between E_{x} and E_{y} is then simulated by directly solving eq. (5) with the time-dependent NA couplings and eigen energies being updated every MD time step. An exemplary single trajectory is shown in Supplementary Fig. 1. Averaging of thousands of such trajectories lead to the exponential decay of around 100 fs as shown in the manuscript.

Normalized magnitudes of the FFT of the trajectories for temperatures 10, 77 and 150 K are shown in Supplementary Fig. 2. Distinct and recurring peaks for all temperatures can be observed at 47, 69, 90, around 150-160 meV, and more elusive modes between 110 and 130 meV.

In order to simulate an excited-state microcanonical trajectory on the E_{x} potential surface, we forced excitation of a spin-down electron to the $\overline{e}_{\mathrm{x}}$ orbital and then the whole NV-diamond of the lower symmetry was heated up to 10 K by repeated velocity rescaling. A 5 ps microcanonical trajectory at 10 K was simulated

on the excited E_x state using the Verlet algorithm with a 1 fs time-step. Here, we removed the constraint of the geometrical symmetry and confirmed that the excited-state structure at each moment is in the C_1 symmetry. The resulting $\overline{e}_x$ and $\overline{e}_y$ orbitals were always non-degenerated as typically shown in the left part of Supplementary Fig. 10. A few thousands of initial conditions were sampled from the long MD trajectory on the E_x state to obtain a converged dephasing function defined as

$$D(t) = \exp[-\int_0^t d\tau_1 \int_0^{\tau_1} d\tau_2 C(\tau_2)] \quad (7)$$

with a time correlation function of energy difference between two focused states $C(t) = < \Delta E(t) \Delta E(0) >$. In the current case, the time-dependent difference between the two potential energies of the E_x and E_y states along the excited-state MD trajectory was used. The dephasing time at 10 K corresponding to τ_2 was deduced by fitting the dephasing function $D(t)$ with a single exponential function, yielding a value of 8 ps, in close agreement with the experimental value of 14 ps. Please note that this value only represents a rough estimate due to the short simulation time.

Supplementary references

1 Ishioka, K., Hase, M., Kitajima, M. & Petek, H. Coherent optical phonons in diamond. *Appl. Phys. Lett.* **89**, 231916 (2006).

2 Davies, G. Vibronic spectra in diamond. *J. Phys. C* **7**, 3797-3809 (1974).

3 Jaumot, J., de Juan, A. & Tauler, R. MCR-ALS GUI 2.0: New features and applications. *Chemometr. Intell. Lab.* **140**, 1-12 (2015).

4 Plakhotnik, T., Doherty, M. W. & Manson, N. B. Electron-phonon processes of the nitrogen-vacancy center in diamond. *Phys. Rev. B* **92**, 081203 (2015).

5 Fu, K. M. *et al.* Observation of the dynamic Jahn-Teller effect in the excited states of nitrogen-vacancy centers in diamond. *Phys. Rev. Lett.* **103**, 256404 (2009).

6 Abtew, T. A. *et al.* Dynamic Jahn-Teller effect in the NV^- center in diamond. *Phys. Rev. Lett.* **107**, 146403 (2011).

7 Lenef, A. *et al.* Electronic structure of the N-V center in diamond: Experiments. *Phys. Rev. B* **53**, 13427-13440 (1996).

8 Hyeon-Deuk, K. & Prezhdo, O. V. Time-Domain ab Initio Study of Auger and Phonon-Assisted Auger Processes in a Semiconductor Quantum Dot. *Nano Lett.* **11**, 1845-1850 (2011).

9 Hyeon-Deuk, K. & Prezhdo, O. V. Multiple Exciton Generation and Recombination Dynamics in Small Si and CdSe Quantum Dots: An Ab Initio Time-Domain Study. *ACS Nano* **6**, 1239-1250 (2012).

10 Hyeon-Deuk, K., Kobayashi, Y. & Tamai, N. Evidence of phonon-assisted Auger recombination and multiple exciton generation in semiconductor quantum dots revealed by temperature-dependent phonon dynamics. *J. Phys. Chem. Lett.* **5**, 99-105 (2014).
